# Complete Reference Genome Assembly for *Commensalibacter* sp. Strain AMU001, an Acetic Acid Bacterium Isolated from the Gut of Honey Bees

**DOI:** 10.1128/MRA.01459-18

**Published:** 2019-01-03

**Authors:** Stefanos Siozios, Josephine Moran, Mary Chege, Gregory D. D. Hurst, Juan C. Paredes

**Affiliations:** a Institute of Integrative Biology, Faculty of Health and Life Sciences, University of Liverpool, Liverpool, United Kingdom; b International Centre of Insect Physiology and Ecology (ICIPE), Nairobi, Kenya; Broad Institute of MIT and Harvard University

## Abstract

We report here the genome sequence of a Commensalibacter sp. strain (AMU001) isolated from honey bees (Apis mellifera) from Seychelles. By combining long- and short-read sequencing technologies, we produced the first complete reference genome assembly for the Commensalibacter genus. We anticipate that this will aid future comparative and functional genomic studies.

## Abstract (precis)

We report here the genome sequence of a Commensalibacter sp. strain (AMU001) isolated from honey bees (Apis mellifera) from Seychelles.

## ANNOUNCEMENT

Acetic acid bacteria (family *Acetobacteraceae*) represent important components of the gut microbial community in many insect taxa that feed on a sugar diet (1). Commensalibacter is a newly described genus within this family (type species, Commensalibacter intestini). It was previously identified as a major component of the Drosophila gut microbiota (2, 3), where it is associated with gut innate immune homeostasis (4). In honey bees, Commensalibacter sp. (also referred to as “Alpha 2.1”) has been reported as a core member of the gut microbiota (5, 6) observed mainly in the midgut and hindgut (7). Currently, draft genome sequences are available for three Commensalibacter isolates (GenBank database, October 2018) (8–10). Here, we combined Oxford Nanopore and Illumina sequencing technologies to produce the first complete and closed genome assembly of Commensalibacter sp. strain AMU001, which was isolated from a Seychelles population of honey bees.

A dominant Commensalibacter phylotype was identified in the gut microbiota from a Seychelles population of honey bees, and a cloned strain was isolated from gut homogenates on de Man-Rogosa-Sharpe (MRS) agar medium. High-molecular-weight DNA was extracted using a phenol-chloroform extraction method (11). A Nanopore sequencing library was prepared using the Rapid Sequencing (SQK-RAD004) protocol (Oxford Nanopore), with slight modifications of the manufacturer’s protocol; in total, 3 μg of DNA were used as input, and library loading beads were omitted. Sequencing was performed on a FLO-MIN106 R9 MinION flow cell. Raw Nanopore signals were live base-called using the processing pipeline implemented in MinKNOW software version 18.01.6 (Oxford Nanopore). Illumina sequencing was performed by MicrobesNG (Birmingham, AL) using the Nextera XT library prep protocol on a MiSeq platform (Illumina, San Diego, CA, USA), and reads were adapter trimmed using Trimmomatic 0.30, with a sliding window quality cutoff of Q15 (12). A *de novo* hybrid assembly of Nanopore and Illumina reads was performed using the Unicycler pipeline version 0.4.5 under the normal mode (13). We assessed the quality of the final assembly by estimating the fraction of interrupted open reading frames (ORFs) using the Ideel method (https://github.com/mw55309/ideel) (14). Additionally, we checked for potential misassemblies by remapping the raw reads on the final assembly and visually inspecting for inconsistencies.

The final assembly of the Commensalibacter strain AMU001 consists of a circular chromosome of 2,013,417 bp (GC content, 37.9%), with an additional circular plasmid of 10,660 bp (GC content, 31.3%). The average depths of coverage of the Nanopore reads were 67× and 835× for the chromosome and the plasmid, respectively. The Illumina data had a relatively uniform coverage of ∼179×. Genome annotation using the NCBI Prokaryotic Genome Annotation Pipeline (15) identified 1,764 protein-coding sequences (CDSs) on the main chromosome and 8 CDSs on the plasmid. The fraction of interrupted ORFs was estimated to about 2% (41 genes with length <80% of the length of their top hit in UniProt database). Finally, 4 rRNA operons and 51 tRNA genes were identified in the main chromosome.

The Commensalibacter sp. genome sequence reported here represents the second for a honey bee-associated Commensalibacter strain and the fourth genome reported for the genus. More importantly, is the first complete and closed genome sequence for the genus and thus represents a valuable reference for further comparative and functional genomic studies.

### Data availability.

The complete genome sequences of the Commensalibacter strain AMU001 main chromosome and its plasmid have been deposited in GenBank under the accession numbers CP033087 and CP033088, respectively (BioProject accession number PRJNA495947). The raw reads are available from the NCBI Sequence Read Archive (SRA) under the accession number SRP166292.
